# Supplementary material for: Triad influence on the detection of crime in Hong Kong
Source: PLoS One. 2024 Feb 28;19(2):e0297145. doi: 10.1371/journal.pone.0297145 (PMC10901352; doi:10.1371/journal.pone.0297145)
Supplement: S1 Appendix — (PDF) [file pone.0297145.s001.pdf]

Appendix 1: Data envelopment analysis (DEA) models on policing services (updated from Barros and Alves [1])

Paper	Method	Units	Inputs	Outputs
Thanassoulis [2]	DEA-CCR	41 police forces of England and Wales, 1992–93	(1) Number of police officers employed;<br>(2) The number of violent crimes;<br>(3) Burglary; and<br>(4) Other crime recorded.	(1) Clear-up rates of violent crime;<br>(2) Clear-up rates of burglary; and<br>(3) Clear-up rates of other crimes recorded.
Carrington, Puthucheary [3]	DEA-CCR and DEA-BCC. Two stages with Tobit model	163 police patrols of New South Wales, Australia, 1994–95.	(1) Number of police officers;<br>(2) The number of civilian officers; and<br>(3) The number of police cars.	(1) Number of offences;<br>(2) number of arrests;<br>(3) Summons;<br>(4) Major car accidents recorded; and<br>(5) Kilometres travelled by police cars.
Nyhan and Martin [4]	DEA-CCR and BCC	36 county and municipal police forces in the US.	(1) Total department costs; and (2) Total personnel (sworn officers and civilians).	(1) The sum of violent and property crimes per 1,000 population; (2) The average response time (from receipt of call to arrival); (3) The clear-up rates resulting from criminal investigations; and<br>(4) The percentage of the sum of violent and property crime clear-ups.
Drake and Simper [5]	DEA and multiple discriminant analysis	English and Wales police forces	(1) Employment costs;<br>(2) The premises-related expenses (the sum of all premises expenses including daily running costs; repair and maintenance);<br>(3) Transport-related expenses; and (4) Capital and other costs (including capital	(1) Clear-up rate;<br>(2) the total number of traffic offences; and<br>(3) the number of breathalyser tests administered

			financing cost and all those costs associated with equipment bought for internal use; such as IT; communications, furniture; contracts-in and contracts-out).	
Drake and Simper [6]	DEA-technical and allocative scores. In a second stage, they divide the police forces in each year into three expenditure groups and with a one-way ANOVA, they investigate the differences between the groups. Later, they estimate the Spearman correlations between overall efficiency and output variables	England and Wales police forces	Same as in Drake & Simper (2000)	Same as Drake & Simper (2000)
Sun [7]	DEA-CCR and BCC with window analysis. In the second stage, a multiple regression is employed to analyse external factors: location, jurisdiction area, population and the proportion of young people living in the area	14 Taipei municipal police precincts,<br>1994–96	(1) The number of police officers;<br>(2) Burglaries;<br>(3) Offences (felonious and violent); and<br>(4) Other crimes.	(1) Burglary clear-ups;<br>(2) Offence clear-ups; and<br>(3) Other crime clear-ups.
Drake and Simper [8]	Compare econometric cost frontier scores with DEA scores	England and Wales police forces	(1) Labour price;<br>(2) Premises prices;	(1) Clear-up rates;

			(3) Transport prices; and<br>(4) Capital price.	(2) The number of traffic offences; and<br>(3) Breathalyser tests administered.
Diez-Ticio and Mancebon [9]	DEA allowing for heterogeneity. In the second stage, they regress the scores by the qualification level of police officers and the proportions of sworn and nonsworn police officers	Spanish police forces at regional level in 1995	(1) The number of police officers;<br>(2) Vehicles; and<br>(3) Population	(1) Clear-up rate.
Drake and Simper [10]	CCR-DEA and free disposal hull (FDH).<br>In the second stage, a Tobit model is used to regress the efficiency scores in contextual variables: population, criminal offences recorded, and 6 geographical dummies	England and Wales police forces	Same as in Drake & Simper (2000)	Same as Drake & Simper (2000)
Drake and Simper [11]	Input oriented DEA-CCR and BCC	England and Wales police forces	(1) Total staff costs;<br>(2) Transport costs; and<br>(3) Capital and other costs.	(1) The number of complaints per officer;<br>(2) The average number of days lost (due to absenteeism) per officer;<br>(3) The number of crimes solved;<br>(4) The number of emergency calls to stations answered; and (5) The number of breathalyser tests.

Hughes and Yaisawarng [12]	DEA-BCC	161 police patrol districts in New South Wales, Australia, 1995–1996	(1) Number of police;<br>(2) Number of civilians;<br>(3) Number of cars;<br>(4) Number of personal computers; and<br>(5) Size of area of station accommodation.	(1) Number of incidents (excl. major car accidents);<br>(2) Number of charges;<br>(3) Number of summons served;<br>(4) Number of major car accidents;<br>(5) Kilometres travelled by police cars; and<br>(6) Intelligence reports.
Drake and Simper [13]	Two stages input oriented DEA-CCR. In the second stage, input slacks are regressed on a set of environmental factors based on a Tobit model.	41 England and Wales police forces	(1) Number of burglaries;<br>(2) Number of vehicle crimes;<br>(3) Number of robberies; and<br>(4) Net budget revenue.	(1) Total offenses cleared; and<br>(2) Total days lost to sickness.
Drake and Simper [14]	DEA and stochastic output distance frontier (SODF)	38 England and Wales police forces	(1) Number of violent crimes against persons;<br>(2) Number of sexual offences;<br>(3) Number of robberies;<br>(4) Number of burglaries of dwelling;<br>(5) Number of theft of a motor vehicle; and<br>(6) Number of theft from a vehicle.	(1) Number of violent crimes against persons cleared;<br>(2) Number of sexual offences cleared;<br>(3) Number of robberies cleared;<br>(4) Number of burglaries of dwelling cleared;<br>(5) Number of theft of a motor vehicle cleared; and<br>(6) Number of theft from a vehicle cleared.

Moore Moore, Nolan [15]	DEA using panel data.	48 police districts in the US, 1993-1998	(1) Number of sworn officers; and<br>(2) Number of support staff.	(1) Crime index for city.
Barros [16]	Input-oriented DEA which allows the estimation of Malmquist productivity index	Portuguese Public Security Police in the city of Lisbon	(1) Number of thefts;<br>(2) Number of burglaries;<br>(3) Number of car robberies; and<br>(4) Number of drug-related crimes.	(1) Clear-ups of thefts and burglaries;<br>(2) Clear-ups of car robberies;<br>(3) Clear-ups of drug-related crimes;<br>(4) Search operations;<br>(5) Traffic-stop operations; and<br>(6) Minor offences.
Goltz [17], Goltz [18]	Input-oriented DEA-BCC	113 municipal and county police departments in Florida, the US, 2005	(1) Total police budget.	(1) Calls for service;<br>(2) Total index crimes;<br>(3) Total arrests; and<br>(4) Total traffic citations.
Verma and Gavirneni [19]	DEA-CCR and BCC and super efficiency DEA allowing a comparison of one selected DMU with all other DMUs present.	Indian police forces in 1997	(1) Total expenditure in crores of Rupees;<br>(2) Number of police officers;<br>(3) Number of investigating officers; and<br>(4) Total number of investigated cases.	(1) Number of persons arrested;<br>(2) Number of persons charge sheeted;<br>(3) Number of persons convicted; and<br>(4) Number of trials completed.
Barros [20]	DEA-CCR and BCC with window analysis. In the second stage, a Tobit regression is employed to analyse external factors: unemployment, slums,	Portuguese Public Security Police in the city of Lisbon – 33 precincts	(1) Number of police officers;<br>(2) Cost of labour;<br>(3) Number of cars;<br>(4) Other costs;<br>(5) Number of thefts;<br>(6) Number of burglaries;	(1) Clear-up rate of theft and burglary;<br>(2) Clear-up rate of stolen cars;<br>(3) Clear-up rate of drug crimes;<br>(4) Number of raids;<br>(5) Number of stop operations; and

	location and number of immigrants.		(7) Number of car robberies; and<br>(8) Number of drug-related crimes.	(6) Number of minor offences.
García-Sánchez [21]	Two stages DEA-CCR and BCC. In the second stage, a logistic regression is employed to analyse explanatory factors: Population, clear-up rates (property crimes, crimes against persons, crimes against sexual freedom and indemnity, and remaining crimes), ratio of arrest and solved crimes (property crimes, crimes against persons, crimes against sexual freedom and indemnity, and remaining crimes)	Spanish National Police Force	(1) Number of offences; and<br>(2) Number of misdemeanours.	(1) Number of individuals arrested in offences; and<br>(2) numbers of individuals arrested in misdemeanours
			(3) Number of offences; and<br>(4) Number of misdemeanours.	(3) Number of offences solved; and<br>(4) Number of misdemeanours solved.
			(5) Number of offences solved; and<br>(6) Number of misdemeanours solved.	(5) Number of individuals arrested in offences; and<br>(6) Numbers of individuals arrested in misdemeanours.
Gorman and Ruggiero [22]	Multiple-stage DEA. In the second stage, a regression is employed to analyse environmental factors: single mothers, population, poverty,	49 state police forces in the US	(1) Number of sworn officers;<br>(2) Number of other employees; and<br>(3) Number of vehicles.	(1) Number of murders;<br>(2) Number of other violent crimes; and<br>(3) Number of total property crimes.

	population per sq. mile, labour force.<br>In the third stage, the environmental cost index is incorporated into the DEA model.			
García-Sánchez [23]	DEA-CCR and BCC	Local police in Spain	(1) Total staff; and<br>(2) Number of vehicles.	(1) Number of kilometres travelled by police vehicles;<br>(2) Number of those arrested taken before the court;<br>(3) Number of objects recovered;<br>(4) Number of interventions made;
				(5) Number of accusations formulated;<br>(6) Number of vehicles removed from the public highway;<br>(7) Number of breathalyzer tests carried out;<br>(8) Number of accident reports drawn up.
Wu, Chen [24]	Multiple-stage DEA. In the second stage, a regression is employed to analyse the effect of degrees of urbanization on policing efficiency.<br>In the third stage, the BCC model incorporates the	22 police districts in Taiwan,<br>1994–96	(1) Labour cost;<br>(2) General operating costs; and<br>(3) Equipment purchasing costs.	(1) The number of burglaries cleared-up; (2) Number of violent crimes cleared up; (3) Number of other crimes cleared up; (4) Number of road traffic accidents resulting in death or serious injury; (5) Number of general and special services in response to requests or pleas by precinct residents (the number of emergency calls

	environmental factors into the equation: the number of public housing units; population; population per square kilometer; percentage of unemployed residents; level of education; percentage of residents aged 15 and above with a college degree or graduate education; and average household yearly revenue			answered is included in this output item); and (6) Residents' satisfaction with the public security of their communities.
Akdogan [25]	DEA-CCR and BCC	19 police stations in Ankara, 2006	(1) The number of personnel;<br>(2) The number of police vehicle;<br>(3) The population of the precinct;<br>(4) The area of the precinct (meter square);<br>(5) The number of critical entities in the precinct (such as schools, hospitals);<br>(6) The number of incoming documents (both judicial and managerial);<br>(7) The number of incidents occurred in the precinct.	(1) The number of processed judicial and managerial documents;<br>(2) The number of outgoing documents; and<br>(3) The number of solved incidents.
Asmild, Paradi [26]	Input oriented DEA-BCC. Some environmental factors (population density, length of the roads and	16 police units	(1) Labour force (number of personnel).	(1) Number of arrests,<br>(2) Number of other worked cases and the case clearance rate;
				(3) Percentage of time available;

	average family income) were included in the models.			(4) Number<br>of calls answered and the inverse of the response time;
				(5) The inverse of the crime rate;<br>(6) Percentage of time on patrol and the number of times people are stopped and interviewed.
Ferrandino [27]	Output-oriented DEA with a pooled approach, which analyses comparative technical efficiency within and between agencies over a 3-year time period.	Campus and municipal Police in Florida, 2008-2010	(1) Total sworn officers.	(1) Traffic citations;<br>(2) Total arrests;<br>and<br>(3) Total index crimes reported.
Verschelde and Rogge [28]	'Benefit-of-the-Doubt' (BoD) model, an adjusted version of DEA, which allows the construction of a perceived effectiveness score by endogenously weighting the citizen satisfaction with the multiple aspects of policing with no inputs	Community oriented local police forces in Belgium	(1) A 'dummy input' equal to one.	(1) Citizen satisfaction on community policing;<br>(2) Reception of citizens;<br>(3) Intervention,<br>aid to victims;<br>(4) Local investigations and detections; and<br>(5) Maintenance of public order.
Aristovnik, Seljak [29]	Three-stage DEA. In the second stage, a regression using ordinary least squares is	11 police directorates in Slovenia, 2010	(1) Number of criminal offenses;<br>(2) Number of active population/1000 inhabitants (ND);	(1) Number of crime scene<br>Inspections;<br>(2) Number of house searches;

	employed to analyse the effect of non-discretionary (uncontrolled) (ND) factors on policing efficiency.<br>In the third stage, the DEA model incorporates the non-discretionary factors into the equation.		(3) Violations of public order regulations;<br>(4) Population size/settlement (ND);<br>(5) Migration increment (ND);	(3) Number of persons held in custody;<br>(4) Number of ordered productions;
			(6) Violations detected during road traffic controls;<br>(7) Number of motor vehicles/1000 inhabitants (ND); and<br>(8) Length of public roads (2008)/km^2 (ND).	(5) Number of examinations (alcohol);<br>(6) Number of temporary confiscations of driving license.
García-Sánchez, Rodríguez-Domínguez [30]	DEA – BCC and adaption of the bootstrapping procedure	52 Spanish provincial police stations, 2001-2009	(1) Number of crimes committed;	(1) Number of arrests;
			(2) The operational efficacy in the previous years; and	(2) The inverse of the variation in the crimes committed; and
			(3) The variation in the number of crimes.	(3) Changes in the population;<br>(4) Economic activity rates; and (5) tourism index over 3 years.
Hadad, Keren [31]	DEA and use of Malmquist Index	13 police stations in the south of Israel, 2008-2011	(1) Number of property crimes;<br>(2) Number of violent crimes;<br>(3) Number of burglaries;<br>(4) Number of traffic accidents; (5) number of traffic accidents with injuries;<br>(6) Total cost in New Israel Shekel;<br>(7) Population size in each precinct in the district; and	(1) Clear-up rate for property crimes;<br>(2) Number of drunken driving cases exposed; and<br>(3) Number of traffic reports (traffic tickets).

			(8) Number of vehicles owned by the residents in the area served by each station.	
Aristovnik, Seljak [32]	Three-stage DEA. In the second stage, a regression using ordinary least squares is employed to analyse the effect of non-discretionary (uncontrolled) (ND) factors on policing efficiency.<br>In the third stage, the DEA model incorporates the non-discretionary factors into the equation.	15 Slovenian police stations, 2010	(1) Posts occupied IT equipment (number of work stations);<br>(2) Police vehicle radio stations<br>Criminal offences (ND);<br>(3) Violations of public order regulations (ND); and<br>(4) Road accidents (ND).	(1) Solved criminal offences;<br>(2) Road accidents involving serious injury;<br>(3) Road accidents involving minor injury;<br>(4) Average response time of police patrols; and<br>(5) Use of instruments of restraint and warning shots.
Raza and Mehmood [33]	DEA and use of Malmquist Index	35 districts across Punjab. 2007-2012.	(1) Total number of expenditure;<br>(2) Number of police officers;<br>(3) Total number of cases to be investigated.	(1) Means 'number of persons charge sheeted;<br>(2) Indicates 'number of trials completed;<br>(3) 'Number of person convicted'.
Hadad, Keren [34]	DEA and use of Malmquist Index	13 police stations in the south of Israel for the years 2008, 2009, 2010, and 2011	(1) Commissioned vehicles (police) and transportation costs;<br>(2) Manpower (numbers and cost);<br>(3) Administration budget for services, perishable, and nonperishable goods;<br>(4) Ongoing maintenance expenses;<br>(5) Custody budget;<br>(6) Special operational budget;	(1) Number of events that have been cleared up (files closed) without investigation;<br>(2) Number of open files (could be sorted by type of criminal act);<br>(3) Number of investigation files (could be sorted by type of criminal act);<br>(4) Number of traffic reports (could be sorted by type of traffic report);

			(7) Budget for supporting volunteers;<br>(8) Size of population;<br>(9) The number of communities in the station's precinct;<br>(10) Number of vehicles in the possession of members of the communities in the station's precinct.	(5) Number of traffic accidents dealt with by the police (could be categorized by severity);<br>(6) Number of police responses to events;<br>(7) Number of arrests;<br>(8) Number of indictments;<br>(9) Number of alcohol tests for drivers.
Parra Domínguez, García Sánchez [35]	Pure technical efficiency was measured using the VRS DEA estimator with the application of bootstrapping	72 countries, 1998-2006	(1) number of police officers	(1) percentage of solved crimes
Chen [36]	DEA and use of Malmquist Index	20 counties/cities in Taiwan for the period 1999–2013	Focusing on public security:<br>(1) Yearly policing expenses (million TWD) per person; and<br>(2) Total number of crime.	Focusing on public security:<br>(1) The number of criminal cases cleared up;<br>(2) The number of burglary cases cleared up; and<br>(3) The number of violent crimes cleared up.
Asif, Shahzad [37]	Output-oriented DEA	71 police stations in the city of Lahore, 2015.	(1) Number of investigators;<br>(2) Total staff;<br>(3) Number of police vehicles; and<br>(4) Operating expenditures.	(1) Average response rate to reported crimes/emergencies;<br>(2) Crime clearance rate;<br>(3) Number of processed judicial and managerial documents; and<br>(4) The number of staff serving at critical entities.
Alda and Dammert [38]	1st stage: Input-oriented DEA	619 Peruvian municipalities	(1) Total number of police per municipality;	(1) Safety index measure, generated by subtracting values of all crimes registered in each municipality from the

	2nd stage: Adjusting inputs to the environmental effects<br>3rd stage: using 2nd stage regression results to predict the total input slacks<br>4th stage: Using adjusted input values and the original output values to re-run the original DEA model		(2) Total number of police vehicles per municipality; and<br>(3) An index measure of technology available for police operations.	municipality with the maximum value of all crimes registered.
Manning and Wong [39]	Output-oriented DEA	18 Hong Kong police districts, 2014	(1) Number of detectives (Model 1,2,3&4);<br>(2) Number of uniformed police (Model 2&4); and<br>(3) Population size (Model 3&4)	(1) Detection rate of violent crimes;<br>(2) Detection rate of burglaries; and<br>(3) Detection rate of other crimes
Wong and Manning [40]	Free Disposable Hull (FDH) approach	18 Hong Kong police districts, 2007-2015	(1) Detective-crime ratio (i.e. the number of detectives in a district divided by the number of crimes reported in a given district); and<br>(2) Uniformed police-crime ratio (i.e. number of uniformed police in a district divided by the number of crimes reported).	(1) Detection rate of violent crimes;<br>(2) Detection rate of Property crimes; and<br>(3) Detection rate of other crimes
Flegl and Gress [41]	Two-stage approach. Four regression analysis models were used to capture the effect of the factors on the calculated efficiency or related dependent variable.	1,730 municipalities in Mexico, 2019	(1) Personnel: such as number of police officers, number of investigating officers or civilian employees;<br>(2) Finance: total staff costs, capital and other costs, total expenditures; and<br>(3) Security infrastructure: such as police cars, technology available for operations, etc.	(1) Number of registered cases and operations;<br>(2) Number of solved cases and operations, usually divided by specific types of crime or level of safety/insecurity

References

1. Barros C, Alves F. Efficiency in crime prevention: a study of Lisbon's police precincts. International Advances in Economic Research. 2005;11:315-28.
2. Thanassoulis E. Assessing police forces in England and Wales using data envelopment analysis. European Journal of Operational Research. 1995;87(3):641-57.
3. Carrington R, Puthucheary N, Rose D, Yaisawarng S. Performance measurement in government service provision: the case of police services in New South Wales. Journal of Productivity Analysis. 1997;8(4):415-30.
4. Nyhan R, Martin L. Assessing the performance of municipal police services using data envelopment analysis: an exploratory study. State and Local Government Review. 1999;31(1):18-30.
5. Drake L, Simper R. Productivity estimation and the size-efficiency relationship in English and Welsh police forces: An application of data envelopment analysis and multiple discriminant analysis. International Review of Law and Economics. 2000;20(1):53-73.
6. Drake L, Simper R. The economic evaluation of policing activity: an application of a hybrid methodology. European Journal of Law and Economics. 2001;12(3):173-92.
7. Sun S. Measuring the relative efficiency of police precincts using data envelopment analysis. Socio-Economic Planning Sciences. 2002;36(1):51-71.
8. Drake L, Simper R. X-efficiency and scale economies in policing: a comparative study using the distribution free approach and DEA. Applied Economics. 2002;34(15):1859-70.
9. Diez-Ticio A, Mancebon M. The efficiency of the Spanish police service: an application of the multiactivity DEA model. Applied Economics. 2002;34(3):351-62.
10. Drake L, Simper R. The measurement of English and Welsh police force efficiency: A comparison of distance function models. European Journal of Operational Research. 2003;147(1):165-86.
11. Drake L, Simper R. The economics of managerialism and the drive for efficiency in policing. Managerial and Decision Economics. 2004;25(8):509-23.
12. Hughes A, Yaisawarng S. Sensitivity and dimensionality tests of DEA efficiency scores. European Journal of Operational Research. 2004;154(2):410-22.
13. Drake L, Simper R. The measurement of police force efficiency: An assessment of U.K. Home Office police. Contemporary Economic Policy. 2005;23(4):465-82.
14. Drake L, Simper R. Police efficiency in offences cleared: An analysis of English "Basic Command Units". International Review of Law and Economics. 2005;25(2):186-208.
15. Moore A, Nolan J, Segal G. Putting out the trash: Measuring municipal service efficiency in US cities. Urban Affairs Review. 2005;41(2):237-59.
16. Barros C. Productivity growth in the Lisbon Police Force. Public Organization Review. 2006;6(1):21-35.
17. Goltz J. Police Organizational Performance in the State of Florida: Confirmatory Analysis of the Relationship of the Environment and Design Structure to Performance. Central Florida: University of Central Florida; 2006.
18. Goltz J. Determinants of performance of police organisations in the state of Florida: an evidence-based confirmatory approach. International Journal of Public Policy. 2008;3(5-6):419-29.
19. Verma A, Gavirneni S. Measuring police efficiency in India: an application of data envelopment analysis. Policing: An International Journal of Police Strategies & Management. 2006;29(1):125-45. doi: doi:10.1108/13639510610648520.
20. Barros C. The city and the police force: analysing relative efficiency in city police precincts with data envelopment analysis. International Journal of Police Science & Management. 2007;9(2):164-82.
21. García-Sánchez I-M. Evaluating the effectiveness of the Spanish police force through data envelopment analysis. European Journal of Law and Economics. 2007;23(1):43-57.
22. Gorman MF, Ruggiero J. Evaluating US state police performance using data envelopment analysis. International Journal of Production Economics. 2008;113(2):1031-7.
23. García-Sánchez I-M. Measuring the efficiency of local police force. European Journal of Law and Economics. 2009;27(1):59-77.
24. Wu T-H, Chen M-S, Yeh J-Y. Measuring the performance of police forces in Taiwan using data envelopment analysis. Evaluation and Program Planning. 2010;33(3):246-54.
25. Akdogan H. The efficiency of police stations in the city of Ankara: an application of data envelopment analysis. Policing. 2012;35(1):25-38. PubMed PMID: 923392309.
26. Asmild M, Paradi J, Pastor J. DEA based models for reallocations of police personnel. OR Spectrum. 2012;34(4):921-41.
27. Ferrandino J. The Comparative Technical Efficiency of Florida Campus Police Departments. Criminal Justice Review. 2012. doi: 10.1177/0734016812442684.
28. Verschelde M, Rogge N. An environment-adjusted evaluation of citizen satisfaction with local police effectiveness: Evidence from a conditional Data Envelopment Analysis approach. European Journal of Operational Research. 2012;223(1):214-25.
29. Aristovnik A, Seljak J, Mencinger J. Relative efficiency of police directorates in Slovenia: A non-parametric analysis. Expert Systems with Applications. 2013;40(2):820-7.
30. García-Sánchez I, Rodríguez-Domínguez L, Parra Domínguez J. Evaluation of the efficacy and effectiveness of the Spanish security forces. European Journal of Law and Economics. 2013;36(1):57-75.
31. Hadad Y, Keren B, Hanani MZ. Combining data envelopment analysis and Malmquist Index for evaluating police station efficiency and effectiveness. Police Practice and Research. 2013;16(1):5-21.
32. Aristovnik A, Seljak J, Mencinger J. Performance measurement of police forces at the local level: A non-parametric mathematical programming approach. Expert Systems with Applications. 2014;41(4, Part 2):1647-53.
33. Raza SH, Mehmood B. Efficiency Differences among Law Enforcing Units in Punjab, Pakistan: Application of Data Envelopment Analysis. Pakistan Journal of Applied Economics. 2014;24(1):17-37.
34. Hadad Y, Keren B, Hanani MZ. Combining data envelopment analysis and Malmquist Index for evaluating police station efficiency and effectiveness. Police Practice and Research. 2015;16(1):5-21. doi: 10.1080/15614263.2013.845945.
35. Parra Domínguez J, García Sánchez IM, Rodríguez Domínguez L. Relationship between police efficiency and crime rate: a worldwide approach. European Journal of Law and Economics. 2015;39(1):203-23. doi: 10.1007/s10657-013-9398-8.
36. Chen CC. Measuring departmental and overall regional performance: applying the multi-activity DEA model to Taiwan′s cities/counties. Omega. 2017;67:60-80. doi: https://doi.org/10.1016/j.omega.2016.04.002.
37. Asif M, Shahzad M, Awan MU, Akdogan H. Developing a structured framework for measuring police efficiency. The International Journal of Quality & Reliability Management. 2018;35(10):2119-35. doi: http://dx.doi.org/10.1108/IJQRM-04-2017-0067. PubMed PMID: 2138610529.
38. Alda E, Dammert L. Weathering the Storm! The effects of the external environment on police efficiency in Peru. Policing. 2019;42(6):1124-40. doi: http://dx.doi.org/10.1108/PIJPSM-03-2019-0033. PubMed PMID: 2315894318.
39. Manning M, Wong GTW. Economic effeciency and the detection of crime: A case study of Hong Kong Policing. In: Wortley R, Sidebottom A, Laycock G, Tilley N, editors. Routledge handbook of crime science. London: Routledge; 2019. p. 350-66.
40. Wong GTW, Manning M. Enhancing Police Efficiency in Detecting Crime in Hong Kong. Crime, Law and Social Change. 2022;78(3):321-55. doi: 10.1007/s10611-022-10027-0.
41. Flegl M, Gress ESH. A two-stage Data Envelopment Analysis model for investigating the efficiency of the public security in Mexico. Decision Analytics Journal. 2023;6:100181. doi: https://doi.org/10.1016/j.dajour.2023.100181.
