# Supplementary material for: Triad influence on the detection of crime in Hong Kong
Source: PLoS One. 2024 Feb 28;19(2):e0297145. doi: 10.1371/journal.pone.0297145 (PMC10901352; doi:10.1371/journal.pone.0297145)
Supplement: S2 Appendix — (PDF) [file pone.0297145.s002.pdf]

Appendix 2. Window analysis of efficiency in crime detection across all districts

District	Year	Efficiency score		
		DEA-CCR	DEA-BCC	Scale
1. Central	2007	91.8	100.0	91.8
2. Central	2008	72.4	83.7	86.5
3. Central	2009	81.1	97.0	83.6
4. Central	2010	59.0	74.0	79.7
5. Central	2011	63.4	82.2	77.1
6. Central	2012	73.0	92.0	79.3
7. Central	2013	62.7	82.2	76.3
8. Central	2014	74.8	86.1	86.9
9. Central	2015	67.1	80.8	83.0
10. Central	2016	78.5	100.0	78.5
11. Central	2017	65.8	79.5	82.8
12. Eastern	2007	95.0	99.1	95.9
13. Eastern	2008	87.2	89.0	97.9
14. Eastern	2009	**100.0**	**100.0**	**100.0**
15. Eastern	2010	97.0	97.5	99.5
16. Eastern	2011	98.8	99.1	99.7
17. Eastern	2012	79.2	79.6	99.5
18. Eastern	2013	78.2	78.6	99.5
19. Eastern	2014	83.5	83.9	99.5
20. Eastern	2015	74.9	75.5	99.2
21. Eastern	2016	72.6	72.8	99.7
22. Eastern	2017	71.9	72.3	99.5
23. Kowloon City	2007	60.7	61.0	99.6
24. Kowloon City	2008	64.2	64.4	99.6
25. Kowloon City	2009	58.1	58.6	99.1
26. Kowloon City	2010	56.1	56.4	99.6
27. Kowloon City	2011	49.6	50.0	99.1
28. Kowloon City	2012	54.7	55.2	99.1
29. Kowloon City	2013	45.3	45.7	99.2
30. Kowloon City	2014	41.0	41.3	99.2
31. Kowloon City	2015	48.5	48.7	99.6
32. Kowloon City	2016	54.3	54.7	99.3
33. Kowloon City	2017	54.1	54.3	99.6
34. Kwai Tsing	2007	89.5	97.9	91.4
35. Kwai Tsing	2008	73.7	79.0	93.3
36. Kwai Tsing	2009	81.6	83.4	97.8
37. Kwai Tsing	2010	73.4	75.3	97.4
38. Kwai Tsing	2011	72.2	73.8	97.7
39. Kwai Tsing	2012	71.1	72.5	98.2
40. Kwai Tsing	2013	63.1	64.0	98.6
41. Kwai Tsing	2014	61.7	62.2	99.1
42. Kwai Tsing	2015	57.5	58.0	99.2
43. Kwai Tsing	2016	59.1	59.8	98.9
44. Kwai Tsing	2017	53.7	54.5	98.6
45. Kwun Tong	2007	95.0	99.0	95.9

46.	Kwun Tong	2008	79.9	84.6	94.5
47.	Kwun Tong	2009	78.5	82.9	94.7
48.	Kwun Tong	2010	80.4	84.9	94.7
49.	Kwun Tong	2011	80.0	84.6	94.6
50.	Kwun Tong	2012	78.8	82.8	95.1
51.	Kwun Tong	2013	70.1	74.2	94.4
52.	Kwun Tong	2014	66.2	70.4	94.0
53.	Kwun Tong	2015	67.8	72.9	93.0
54.	Kwun Tong	2016	62.6	68.7	91.1
55.	Kwun Tong	2017	63.8	70.7	90.2
56.	Lantau	2007	28.0	61.8	45.3
57.	Lantau	2008	27.2	43.5	62.7
58.	Lantau	2009	30.3	56.5	53.6
59.	Lantau	2010	25.9	41.7	62.1
60.	Lantau	2011	31.6	45.8	68.9
61.	Lantau	2012	27.4	44.3	61.7
62.	Lantau	2013	32.9	100.0	32.9
63.	Lantau	2014	32.0	86.2	37.1
64.	Lantau	2015	35.6	100.0	35.6
65.	Lantau	2016	30.7	39.1	78.5
66.	Lantau	2017	23.9	30.9	77.5
67.	Mongkok	2007	**100.0**	**100.0**	**100.0**
68.	Mongkok	2008	**100.0**	**100.0**	**100.0**
69.	Mongkok	2009	93.6	95.1	98.4
70.	Mongkok	2010	80.0	80.6	99.3
71.	Mongkok	2011	74.6	74.8	99.8
72.	Mongkok	2012	86.7	87.2	99.5
73.	Mongkok	2013	83.0	83.3	99.7
74.	Mongkok	2014	79.1	79.7	99.2
75.	Mongkok	2015	91.1	92.2	98.8
76.	Mongkok	2016	96.4	100.0	96.4
77.	Mongkok	2017	92.7	96.1	96.5
78.	Sau Mau Ping	2007	65.2	73.0	89.4
79.	Sau Mau Ping	2008	56.6	61.4	92.2
80.	Sau Mau Ping	2009	56.9	63.3	89.9
81.	Sau Mau Ping	2010	62.9	66.5	94.5
82.	Sau Mau Ping	2011	57.0	60.8	93.9
83.	Sau Mau Ping	2012	60.6	64.2	94.4
84.	Sau Mau Ping	2013	54.0	56.6	95.2
85.	Sau Mau Ping	2014	49.1	50.5	97.2
86.	Sau Mau Ping	2015	42.2	43.1	97.9
87.	Sau Mau Ping	2016	44.0	44.7	98.4
88.	Sau Mau Ping	2017	41.8	42.3	98.9
89.	Sha Tin	2007	91.8	92.7	99.1
90.	Sha Tin	2008	77.9	79.1	98.5
91.	Sha Tin	2009	84.9	85.1	99.7
92.	Sha Tin	2010	82.3	82.8	99.3
93.	Sha Tin	2011	90.8	91.6	99.1

94.	Sha Tin	2012	86.9	87.8	99.0
95.	Sha Tin	2013	75.0	76.1	98.6
96.	Sha Tin	2014	78.0	79.3	98.3
97.	Sha Tin	2015	78.5	80.2	97.9
98.	Sha Tin	2016	70.4	71.8	98.1
99.	Sha Tin	2017	62.2	63.6	97.8
100.	Sham Shui Po	2007	79.0	79.4	99.6
101.	Sham Shui Po	2008	81.0	81.7	99.2
102.	Sham Shui Po	2009	87.4	87.4	100.0
103.	Sham Shui Po	2010	83.9	84.2	99.7
104.	Sham Shui Po	2011	74.2	74.7	99.3
105.	Sham Shui Po	2012	76.3	77.0	99.1
106.	Sham Shui Po	2013	70.5	70.8	99.6
107.	Sham Shui Po	2014	62.7	63.3	98.9
108.	Sham Shui Po	2015	66.4	67.4	98.6
109.	Sham Shui Po	2016	68.9	70.5	97.7
110.	Sham Shui Po	2017	71.0	73.3	96.9
111.	Tai Po	2007	88.1	90.9	96.9
112.	Tai Po	2008	90.9	93.5	97.2
113.	Tai Po	2009	95.7	98.0	97.7
114.	Tai Po	2010	87.7	89.1	98.5
115.	Tai Po	2011	86.1	87.1	98.8
116.	Tai Po	2012	77.0	77.4	99.5
117.	Tai Po	2013	70.4	71.2	98.9
118.	Tai Po	2014	63.2	63.7	99.2
119.	Tai Po	2015	68.0	68.6	99.2
120.	Tai Po	2016	62.9	65.0	96.8
121.	Tai Po	2017	59.9	62.9	95.2
122.	Tsuen Wan	2007	51.9	53.3	97.4
123.	Tsuen Wan	2008	52.9	53.3	99.1
124.	Tsuen Wan	2009	54.0	54.8	98.5
125.	Tsuen Wan	2010	49.6	51.0	97.3
126.	Tsuen Wan	2011	53.1	54.8	97.0
127.	Tsuen Wan	2012	50.5	52.6	96.1
128.	Tsuen Wan	2013	50.9	52.7	96.6
129.	Tsuen Wan	2014	48.1	49.7	96.6
130.	Tsuen Wan	2015	51.0	52.4	97.3
131.	Tsuen Wan	2016	44.0	46.4	95.0
132.	Tsuen Wan	2017	37.5	38.9	96.4
133.	Tuen Mun	2007	72.9	73.1	99.7
134.	Tuen Mun	2008	81.5	82.2	99.1
135.	Tuen Mun	2009	80.5	81.1	99.2
136.	Tuen Mun	2010	72.5	72.9	99.5
137.	Tuen Mun	2011	80.9	81.1	99.8
138.	Tuen Mun	2012	69.3	69.3	100.0
139.	Tuen Mun	2013	61.9	62.4	99.1
140.	Tuen Mun	2014	61.6	63.3	97.3
141.	Tuen Mun	2015	58.8	61.5	95.7

142.	Tuen Mun	2016	63.2	65.6	96.3
143.	Tuen Mun	2017	55.7	58.6	95.1
144.	Wanchai	2007	76.6	80.1	95.6
145.	Wanchai	2008	84.2	89.7	93.9
146.	Wanchai	2009	91.5	97.3	94.0
147.	Wanchai	2010	84.4	89.0	94.8
148.	Wanchai	2011	89.6	94.9	94.4
149.	Wanchai	2012	93.9	100.0	93.9
150.	Wanchai	2013	88.0	93.5	94.1
151.	Wanchai	2014	89.0	95.2	93.5
152.	Wanchai	2015	86.5	93.9	92.1
153.	Wanchai	2016	83.7	90.2	92.8
154.	Wanchai	2017	69.3	74.9	92.5
155.	Western	2007	78.7	100.0	78.7
156.	Western	2008	77.1	87.3	88.4
157.	Western	2009	70.1	85.8	81.7
158.	Western	2010	64.4	75.3	85.5
159.	Western	2011	69.1	77.4	89.2
160.	Western	2012	62.0	71.6	86.7
161.	Western	2013	55.6	68.9	80.7
162.	Western	2014	53.9	64.4	83.7
163.	Western	2015	55.9	61.9	90.3
164.	Western	2016	52.8	63.9	82.6
165.	Western	2017	55.8	66.0	84.5
166.	Wong Tai Sin	2007	67.3	70.6	95.2
167.	Wong Tai Sin	2008	72.0	76.1	94.7
168.	Wong Tai Sin	2009	74.4	76.3	97.5
169.	Wong Tai Sin	2010	58.7	60.7	96.6
170.	Wong Tai Sin	2011	56.4	58.0	97.1
171.	Wong Tai Sin	2012	63.8	65.7	97.2
172.	Wong Tai Sin	2013	62.1	62.9	98.7
173.	Wong Tai Sin	2014	54.7	55.3	98.8
174.	Wong Tai Sin	2015	58.2	59.3	98.1
175.	Wong Tai Sin	2016	48.1	49.0	98.3
176.	Wong Tai Sin	2017	44.4	44.8	99.1
177.	Yau Tsim	2007	97.6	99.9	97.7
178.	Yau Tsim	2008	98.0	100.0	98.0
179.	Yau Tsim	2009	90.0	94.1	95.6
180.	Yau Tsim	2010	69.7	73.0	95.4
181.	Yau Tsim	2011	79.7	81.4	97.9
182.	Yau Tsim	2012	82.9	88.4	93.8
183.	Yau Tsim	2013	76.8	80.7	95.1
184.	Yau Tsim	2014	72.6	74.9	96.9
185.	Yau Tsim	2015	80.4	82.4	97.6
186.	Yau Tsim	2016	92.9	100.0	92.9
187.	Yau Tsim	2017	91.1	99.2	91.8
188.	Yuen Long	2007	**100.0**	**100.0**	**100.0**
189.	Yuen Long	2008	94.4	95.4	98.9

190.	Yuen Long	2009	88.5	90.4	97.8
191.	Yuen Long	2010	88.5	89.1	99.4
192.	Yuen Long	2011	85.4	87.2	98.0
193.	Yuen Long	2012	90.5	90.7	99.8
194.	Yuen Long	2013	88.4	88.7	99.7
195.	Yuen Long	2014	84.6	85.4	99.0
196.	Yuen Long	2015	83.3	85.6	97.3
197.	Yuen Long	2016	72.6	78.7	92.3
198.	Yuen Long	2017	65.8	72.0	91.3
		**Mean (SD)**	**69.7 (17.6)**	**74.6 (16.7)**	**93.4 (11.1)**
