# Supplementary material for: Triad influence on the detection of crime in Hong Kong
Source: PLoS One. 2024 Feb 28;19(2):e0297145. doi: 10.1371/journal.pone.0297145 (PMC10901352; doi:10.1371/journal.pone.0297145)
Supplement: S3 Appendix — (PDF) [file pone.0297145.s003.pdf]

Appendix 3. DEA-BCC slack results for each input and output for the DMUs

		Slacks														
		Inputs		Outputs			Recommended target quantity					Potential improvement				
Districts		Year	i_d	i_u	o_vc	o_pc	o_oc	i_d	i_u	o_vc	o_pc	o_oc	i_d	i_u	o_vc	o_pc	o_oc
1.	Central	2007	0.00	0.00	0.00	0.00	0.00	74.22	480.07	256.00	385.00	548.00	0.0%	0.0%	0.0%	0.0%	0.0%
2.	Central	2008	0.00	0.41	52.18	0.00	0.00	79.40	493.75	241.18	368.00	474.00	0.0%	-0.1%	27.6%	0.0%	0.0%
3.	Central	2009	0.00	0.00	35.62	0.00	0.00	77.50	488.32	255.62	430.00	469.00	0.0%	0.0%	16.2%	0.0%	0.0%
4.	Central	2010	0.00	6.70	0.00	0.00	102.92	80.04	489.12	225.00	325.00	450.92	0.0%	-1.4%	0.0%	0.0%	29.6%
5.	Central	2011	0.00	0.00	0.00	0.43	121.07	79.87	494.73	251.00	351.43	490.07	0.0%	0.0%	0.0%	0.1%	32.8%
6.	Central	2012	0.00	10.02	0.00	0.00	51.53	77.96	478.78	252.00	411.00	423.53	0.0%	-2.0%	0.0%	0.0%	13.9%
7.	Central	2013	0.00	0.00	0.00	6.43	131.63	77.45	485.79	233.00	340.43	490.63	0.0%	0.0%	0.0%	1.9%	36.7%
8.	Central	2014	0.00	0.00	0.00	38.34	41.03	79.05	489.68	256.00	371.34	501.03	0.0%	0.0%	0.0%	11.5%	8.9%
9.	Central	2015	0.00	4.55	0.00	0.00	5.16	78.29	482.36	228.00	354.00	408.16	0.0%	-0.9%	0.0%	0.0%	1.3%
10.	Central	2016	0.00	0.00	0.00	0.00	0.00	71.50	466.79	217.00	394.00	398.00	0.0%	0.0%	0.0%	0.0%	0.0%
11.	Central	2017	1.34	0.00	0.00	1.46	0.00	73.52	475.59	194.00	311.46	392.00	-1.8%	0.0%	0.0%	0.5%	0.0%
12.	Eastern	2007	0.00	41.55	0.00	0.00	83.37	119.16	585.35	564.00	845.00	690.37	0.0%	-6.6%	0.0%	0.0%	13.7%
13.	Eastern	2008	0.00	27.32	0.00	0.00	21.48	120.08	602.93	487.00	792.00	591.48	0.0%	-4.3%	0.0%	0.0%	3.8%
14.	Eastern	2009	0.00	0.00	0.00	0.00	0.00	117.25	620.80	479.00	908.00	579.00	0.0%	0.0%	0.0%	0.0%	0.0%
15.	Eastern	2010	0.00	9.11	49.51	0.00	130.60	120.14	621.18	501.51	900.00	602.60	0.0%	-1.4%	11.0%	0.0%	27.7%

16.	Eastern	2011	0.00	6.72	93.71	0.00	156.76	119.73	621.13	504.71	913.00	605.76	0.0%	-1.1%	22.8%	0.0%	34.9%
17.	Eastern	2012	0.00	11.93	0.00	0.00	0.00	119.92	615.18	411.00	723.00	506.00	0.0%	-1.9%	0.0%	0.0%	0.0%
18.	Eastern	2013	0.00	9.23	78.62	0.00	54.84	121.15	621.32	429.62	730.00	504.84	0.0%	-1.5%	22.4%	0.0%	12.2%
19.	Eastern	2014	0.00	10.43	195.54	0.00	99.14	121.99	621.43	480.54	783.00	551.14	0.0%	-1.7%	68.6%	0.0%	21.9%
20.	Eastern	2015	0.00	15.60	192.61	0.00	84.45	124.17	621.72	470.61	713.00	522.45	0.0%	-2.4%	69.3%	0.0%	19.3%
21.	Eastern	2016	0.00	5.40	134.86	0.00	17.23	121.13	621.32	418.86	676.00	461.23	0.0%	-0.9%	47.5%	0.0%	3.9%
22.	Eastern	2017	0.00	10.11	203.76	0.00	96.71	123.22	621.59	453.76	679.00	498.71	0.0%	-1.6%	81.5%	0.0%	24.1%
23.	Kowloon City	2007	0.00	13.95	0.00	72.15	0.00	153.28	608.28	498.00	668.15	681.00	0.0%	-2.2%	0.0%	12.1%	0.0%
24.	Kowloon City	2008	0.00	0.00	250.34	0.00	0.00	152.97	620.38	671.34	702.00	664.00	0.0%	0.0%	59.5%	0.0%	0.0%
25.	Kowloon City	2009	0.00	0.00	123.15	0.00	28.78	151.02	614.22	552.15	629.00	597.78	0.0%	0.0%	28.7%	0.0%	5.1%
26.	Kowloon City	2010	0.00	0.00	199.18	0.00	137.88	153.01	620.28	589.18	615.00	636.88	0.0%	0.0%	51.1%	0.0%	27.6%
27.	Kowloon City	2011	0.00	0.00	103.05	0.00	134.75	151.14	614.58	480.05	537.00	567.75	0.0%	0.0%	27.3%	0.0%	31.1%
28.	Kowloon City	2012	0.00	0.00	196.84	0.00	155.36	151.05	613.93	559.84	592.00	621.36	0.0%	0.0%	54.2%	0.0%	33.3%
29.	Kowloon City	2013	0.00	0.76	0.00	0.00	0.00	150.36	612.44	350.00	477.00	487.00	0.0%	-0.1%	0.0%	0.0%	0.0%
30.	Kowloon City	2014	0.00	5.97	0.00	0.00	0.00	151.91	610.88	338.00	434.00	448.00	0.0%	-1.0%	0.0%	0.0%	0.0%

31. Kowloon City	2015	0.00	13.64	0.00	47.18	0.00	152.98	605.75	379.00	530.18	551.00	0.0%	-2.2%	0.0%	9.8%	0.0%
32. Kowloon City	2016	0.00	0.00	310.68	0.00	0.00	151.85	614.55	633.68	584.00	575.00	0.0%	0.0%	96.2%	0.0%	0.0%
33. Kowloon City	2017	0.00	0.00	318.20	0.00	171.66	154.07	620.24	636.20	596.00	641.66	0.0%	0.0%	100.1%	0.0%	36.5%
34. Kwai Tsing	2007	0.00	0.00	0.00	23.34	116.18	129.44	593.03	715.00	823.34	837.18	0.0%	0.0%	0.0%	2.9%	16.1%
35. Kwai Tsing	2008	0.00	13.11	0.00	0.00	138.64	132.03	587.77	593.00	693.00	739.64	0.0%	-2.2%	0.0%	0.0%	23.1%
36. Kwai Tsing	2009	0.00	0.00	85.47	0.00	0.00	130.50	595.39	591.47	768.00	699.00	0.0%	0.0%	16.9%	0.0%	0.0%
37. Kwai Tsing	2010	0.00	0.00	46.51	0.00	0.00	131.15	595.60	485.51	708.00	589.00	0.0%	0.0%	10.6%	0.0%	0.0%
38. Kwai Tsing	2011	0.00	0.00	43.32	0.00	0.00	133.09	599.74	481.32	708.00	584.00	0.0%	0.0%	9.9%	0.0%	0.0%
39. Kwai Tsing	2012	0.00	1.80	0.00	0.00	0.00	134.83	602.61	454.00	686.00	649.00	0.0%	-0.3%	0.0%	0.0%	0.0%
40. Kwai Tsing	2013	0.00	0.06	0.00	0.00	0.00	135.85	606.62	427.00	617.00	567.00	0.0%	0.0%	0.0%	0.0%	0.0%
41. Kwai Tsing	2014	0.00	0.00	119.23	0.00	0.00	138.56	613.54	490.23	627.00	528.00	0.0%	0.0%	32.1%	0.0%	0.0%
42. Kwai Tsing	2015	0.00	5.25	0.00	0.00	0.00	141.12	615.90	390.00	584.00	538.00	0.0%	-0.8%	0.0%	0.0%	0.0%
43. Kwai Tsing	2016	0.00	11.13	0.00	0.00	0.00	141.94	611.20	440.00	601.00	566.00	0.0%	-1.8%	0.0%	0.0%	0.0%
44. Kwai Tsing	2017	0.00	23.05	0.00	0.00	0.00	144.00	604.06	400.00	543.00	550.00	0.0%	-3.7%	0.0%	0.0%	0.0%
45. Kwun Tong	2007	4.76	50.34	151.90	0.00	408.18	160.29	626.51	980.90	1126.00	1106.18	-2.9%	-7.4%	18.3%	0.0%	58.5%
46. Kwun Tong	2008	7.30	59.57	208.94	0.00	368.40	160.29	626.51	868.94	962.00	998.40	-4.4%	-8.7%	31.7%	0.0%	58.5%

47. Kwun Tong	2009	6.83	60.06	111.23	0.00	355.80	160.29	626.51	839.23	943.00	983.80	-4.1%	-8.7%	15.3%	0.0%	56.7%
48. Kwun Tong	2010	7.02	59.33	53.48	0.00	408.41	160.29	626.51	847.48	965.00	1006.41	-4.2%	-8.7%	6.7%	0.0%	68.3%
49. Kwun Tong	2011	6.96	60.47	143.93	0.00	383.76	160.29	626.51	858.93	962.00	1000.76	-4.2%	-8.8%	20.1%	0.0%	62.2%
50. Kwun Tong	2012	5.94	57.92	180.31	0.00	355.00	160.29	626.51	850.31	942.00	983.00	-3.6%	-8.5%	26.9%	0.0%	56.5%
51. Kwun Tong	2013	7.25	61.83	173.97	0.00	254.86	160.29	626.51	778.97	844.00	891.86	-4.3%	-9.0%	28.8%	0.0%	40.0%
52. Kwun Tong	2014	8.01	64.30	359.39	0.00	327.05	160.29	626.51	802.39	800.00	880.05	-4.8%	-9.3%	81.1%	0.0%	59.1%
53. Kwun Tong	2015	10.07	70.76	325.18	0.00	287.34	160.29	626.51	809.18	829.00	889.34	-5.9%	-10.1%	67.2%	0.0%	47.7%
54. Kwun Tong	2016	13.92	80.70	314.95	0.00	242.42	160.29	626.51	777.95	781.00	840.42	-8.0%	-11.4%	68.0%	0.0%	40.5%
55. Kwun Tong	2017	15.77	86.01	413.43	0.00	217.82	160.29	626.51	820.43	804.00	850.82	-9.0%	-12.1%	101.6%	0.0%	34.4%
56. Lantau	2007	9.30	0.00	0.00	194.32	233.47	72.99	469.46	142.00	325.32	342.47	-11.3%	0.0%	0.0%	148.3%	214.2%
57. Lantau	2008	4.30	0.00	0.00	192.55	292.81	87.30	495.20	154.00	337.55	393.81	-4.7%	0.0%	0.0%	132.8%	289.9%
58. Lantau	2009	7.24	0.00	0.00	213.34	266.17	79.55	481.26	162.00	353.34	377.17	-8.3%	0.0%	0.0%	152.4%	239.8%

59.	Lantau	2010	6.01	0.00	0.00	89.36	245.80	82.70	486.93	131.00	255.36	346.80	-6.8%	0.0%	0.0%	53.8%	243.4%
60.	Lantau	2011	6.72	0.00	0.00	25.26	253.03	81.69	485.11	140.00	233.26	357.03	-7.6%	0.0%	0.0%	12.1%	243.3%
61.	Lantau	2012	9.60	0.00	0.00	29.61	120.75	74.03	471.34	106.00	200.61	252.75	-11.5%	0.0%	0.0%	17.3%	91.5%
62.	Lantau	2013	0.00	0.00	0.00	0.00	0.00	78.51	455.63	141.00	189.00	145.00	0.0%	0.0%	0.0%	0.0%	0.0%
63.	Lantau	2014	2.46	0.00	0.00	17.59	5.46	77.08	457.91	135.00	201.59	170.46	-3.1%	0.0%	0.0%	9.6%	3.3%
64.	Lantau	2015	0.00	0.00	0.00	0.00	0.00	78.85	455.56	131.00	212.00	156.00	0.0%	0.0%	0.0%	0.0%	0.0%
65.	Lantau	2016	0.00	0.00	0.00	0.00	198.95	95.69	502.18	109.00	227.00	338.95	0.0%	0.0%	0.0%	0.0%	142.1%
66.	Lantau	2017	0.00	0.00	0.00	0.00	166.20	93.65	497.18	90.00	171.00	284.20	0.0%	0.0%	0.0%	0.0%	140.8%
67.	Mongkok	2007	0.00	0.00	0.00	0.00	0.00	168.19	609.99	438.00	1064.00	1436.00	0.0%	0.0%	0.0%	0.0%	0.0%
68.	Mongkok	2008	0.00	0.00	0.00	0.00	0.00	167.64	606.73	475.00	1068.00	1418.00	0.0%	0.0%	0.0%	0.0%	0.0%
69.	Mongkok	2009	2.15	0.00	0.00	0.00	0.00	164.26	602.97	533.00	916.00	1255.00	-1.3%	0.0%	0.0%	0.0%	0.0%
70.	Mongkok	2010	7.24	0.00	208.42	0.00	0.00	161.75	610.70	612.42	880.00	941.00	-4.3%	0.0%	51.6%	0.0%	0.0%
71.	Mongkok	2011	7.61	0.00	130.59	0.00	0.00	163.11	615.30	584.59	823.00	918.00	-4.5%	0.0%	28.8%	0.0%	0.0%
72.	Mongkok	2012	2.58	0.00	0.00	0.00	0.00	165.14	606.56	484.00	935.00	1139.00	-1.5%	0.0%	0.0%	0.0%	0.0%
73.	Mongkok	2013	2.03	0.00	0.00	0.00	0.00	166.04	606.74	438.00	892.00	1122.00	-1.2%	0.0%	0.0%	0.0%	0.0%

74.	Mongkok	2014	2.74	0.00	0.00	0.00	0.00	165.22	606.01	426.00	848.00	1071.00	-1.6%	0.0%	0.0%	0.0%	0.0%
75.	Mongkok	2015	6.67	0.00	265.84	0.00	0.00	161.21	605.44	616.84	994.00	1052.00	-4.0%	0.0%	75.7%	0.0%	0.0%
76.	Mongkok	2016	0.00	0.00	0.00	0.00	0.00	158.22	577.36	316.00	1009.00	997.00	0.0%	0.0%	0.0%	0.0%	0.0%
77.	Mongkok	2017	0.00	0.00	0.00	0.00	0.00	159.24	579.68	326.00	972.00	993.00	0.0%	0.0%	0.0%	0.0%	0.0%
78.	Sau Mau Ping	2007	2.80	0.00	0.00	209.26	136.63	132.08	575.76	543.00	714.26	683.63	-2.1%	0.0%	0.0%	41.4%	25.0%
79.	Sau Mau Ping	2008	1.46	0.00	0.00	174.06	34.07	135.38	581.70	474.00	637.06	573.07	-1.1%	0.0%	0.0%	37.6%	6.3%
80.	Sau Mau Ping	2009	2.49	0.00	0.00	142.68	100.42	133.04	577.48	476.00	627.68	602.42	-1.8%	0.0%	0.0%	29.4%	20.0%
81.	Sau Mau Ping	2010	0.00	0.00	0.00	0.00	146.94	136.13	579.05	429.00	621.00	624.94	0.0%	0.0%	0.0%	0.0%	30.7%
82.	Sau Mau Ping	2011	0.00	0.00	0.00	0.00	121.60	135.51	577.09	397.00	561.00	578.60	0.0%	0.0%	0.0%	0.0%	26.6%
83.	Sau Mau Ping	2012	0.00	0.00	0.00	0.00	203.92	134.88	576.67	387.00	594.00	610.92	0.0%	0.0%	0.0%	0.0%	50.1%
84.	Sau Mau Ping	2013	0.00	0.00	0.00	0.00	76.08	136.09	580.02	344.00	533.00	507.08	0.0%	0.0%	0.0%	0.0%	17.7%
85.	Sau Mau Ping	2014	0.00	0.00	143.91	0.00	0.00	137.69	587.26	462.91	481.00	464.00	0.0%	0.0%	45.1%	0.0%	0.0%

86. Sau Mau Ping	2015	0.00	0.00	77.43	0.00	0.00	139.44	591.95	392.43	413.00	417.00	0.0%	0.0%	24.6%	0.0%	0.0%
87. Sau Mau Ping	2016	0.00	0.00	131.29	0.00	93.34	143.90	605.33	409.29	458.00	459.34	0.0%	0.0%	47.2%	0.0%	25.5%
88. Sau Mau Ping	2017	0.00	0.00	149.41	0.00	42.19	145.94	611.68	420.41	441.00	420.19	0.0%	0.0%	55.1%	0.0%	11.2%
89. Sha Tin	2007	0.00	14.90	0.00	0.00	235.02	136.54	614.12	672.00	921.00	798.02	0.0%	-2.4%	0.0%	0.0%	41.7%
90. Sha Tin	2008	0.00	21.06	0.00	0.00	157.97	136.73	608.89	583.00	779.00	711.97	0.0%	-3.3%	0.0%	0.0%	28.5%
91. Sha Tin	2009	0.00	5.68	93.68	0.00	111.47	136.30	623.33	613.68	859.00	710.47	0.0%	-0.9%	18.0%	0.0%	18.6%
92. Sha Tin	2010	0.00	15.04	112.77	0.00	226.74	140.14	623.83	640.77	853.00	753.74	0.0%	-2.4%	21.4%	0.0%	43.0%
93. Sha Tin	2011	0.00	19.04	242.36	0.00	305.81	141.80	624.05	725.36	951.00	834.81	0.0%	-3.0%	50.2%	0.0%	57.8%
94. Sha Tin	2012	0.00	23.51	225.69	0.00	236.57	143.24	624.25	718.69	919.00	820.57	0.0%	-3.6%	45.8%	0.0%	40.5%
95. Sha Tin	2013	0.00	31.65	90.94	0.00	206.95	145.95	624.61	644.94	807.00	760.95	0.0%	-4.8%	16.4%	0.0%	37.4%
96. Sha Tin	2014	0.00	39.63	395.63	0.00	326.72	148.94	625.00	759.63	854.00	838.72	0.0%	-6.0%	108.7%	0.0%	63.8%
97. Sha Tin	2015	0.00	50.40	464.50	0.00	406.06	152.74	625.50	813.50	880.00	898.06	0.0%	-7.5%	133.1%	0.0%	82.5%
98. Sha Tin	2016	0.00	45.91	407.24	0.00	396.26	150.44	625.20	741.24	779.00	823.26	0.0%	-6.8%	121.9%	0.0%	92.8%
99. Sha Tin	2017	0.00	53.41	312.19	0.00	341.01	153.04	625.54	688.19	699.00	775.01	0.0%	-7.9%	83.0%	0.0%	78.6%
100. Sham Shui Po	2007	0.00	0.00	173.85	0.00	0.00	158.18	615.87	710.85	867.00	910.00	0.0%	0.0%	32.4%	0.0%	0.0%

101. Sham Shui Po	2008	1.24	6.55	77.17	0.00	0.00	162.24	622.44	711.17	914.00	974.00	-0.8%	-1.0%	12.2%	0.0%	0.0%
102. Sham Shui Po	2009	0.23	0.00	174.00	0.00	0.00	161.35	623.14	815.00	984.00	1011.00	-0.1%	0.0%	27.1%	0.0%	0.0%
103. Sham Shui Po	2010	0.82	1.33	177.02	0.00	0.00	161.70	623.57	778.02	946.00	985.00	-0.5%	-0.2%	29.5%	0.0%	0.0%
104. Sham Shui Po	2011	2.97	3.90	109.80	0.00	0.00	161.12	624.78	723.80	844.00	857.00	-1.8%	-0.6%	17.9%	0.0%	0.0%
105. Sham Shui Po	2012	2.17	4.62	152.16	0.00	0.00	161.60	623.77	726.16	866.00	898.00	-1.3%	-0.7%	26.5%	0.0%	0.0%
106. Sham Shui Po	2013	2.88	2.21	223.46	0.00	0.00	160.57	625.92	751.46	803.00	796.00	-1.8%	-0.4%	42.3%	0.0%	0.0%
107. Sham Shui Po	2014	4.97	6.67	229.42	0.00	4.43	160.29	626.51	710.42	720.00	706.43	-3.0%	-1.1%	47.7%	0.0%	0.6%
108. Sham Shui Po	2015	6.02	9.12	316.60	0.00	11.62	160.29	626.51	769.60	766.00	753.62	-3.6%	-1.4%	69.9%	0.0%	1.6%
109. Sham Shui Po	2016	6.85	14.32	254.33	0.00	0.00	161.03	624.97	736.33	797.00	806.00	-4.1%	-2.2%	52.8%	0.0%	0.0%
110. Sham Shui Po	2017	10.40	20.23	388.42	0.00	109.76	160.29	626.51	828.42	833.00	844.76	-6.1%	-3.1%	88.3%	0.0%	14.9%
111. Tai Po	2007	0.00	0.00	3.39	0.00	35.99	132.28	588.07	528.39	856.00	702.99	0.0%	0.0%	0.6%	0.0%	5.4%
112. Tai Po	2008	0.00	0.00	187.74	0.00	0.00	133.00	588.65	685.74	870.00	802.00	0.0%	0.0%	37.7%	0.0%	0.0%
113. Tai Po	2009	0.00	0.00	69.35	0.00	0.00	136.65	599.16	663.35	957.00	818.00	0.0%	0.0%	11.7%	0.0%	0.0%
114. Tai Po	2010	0.00	0.00	70.89	0.00	72.50	139.30	605.61	631.89	892.00	769.50	0.0%	0.0%	12.6%	0.0%	10.4%

115. Tai Po	2011	0.00	0.00	80.65	0.00	74.22	141.02	610.19	646.65	885.00	772.22	0.0%	0.0%	14.2%	0.0%	10.6%
116. Tai Po	2012	0.00	0.00	84.84	0.00	0.00	143.94	618.18	640.84	804.00	715.00	0.0%	0.0%	15.3%	0.0%	0.0%
117. Tai Po	2013	0.00	22.76	0.00	0.00	0.00	148.02	606.65	520.00	724.00	755.00	0.0%	-3.6%	0.0%	0.0%	0.0%
118. Tai Po	2014	0.00	18.32	170.63	0.00	37.80	153.27	625.58	638.63	700.00	666.80	0.0%	-2.8%	36.5%	0.0%	6.0%
119. Tai Po	2015	0.00	31.73	195.25	0.00	38.11	158.15	626.22	722.25	772.00	757.11	0.0%	-4.8%	37.0%	0.0%	5.3%
120. Tai Po	2016	2.65	49.96	341.26	0.00	91.39	160.29	626.51	762.26	739.00	755.39	-1.6%	-7.4%	81.1%	0.0%	13.8%
121. Tai Po	2017	5.60	58.15	426.07	0.00	190.68	160.29	626.51	780.07	715.00	770.68	-3.4%	-8.5%	120.4%	0.0%	32.9%
122. Tsuen Wan	2007	0.00	0.00	0.00	0.00	142.96	150.60	603.49	417.00	558.00	598.96	0.0%	0.0%	0.0%	0.0%	31.4%
123. Tsuen Wan	2008	0.00	0.00	0.00	38.88	0.00	152.25	607.67	454.00	562.88	578.00	0.0%	0.0%	0.0%	7.4%	0.0%
124. Tsuen Wan	2009	0.00	0.00	2.03	0.00	0.00	149.04	597.74	404.03	551.00	599.00	0.0%	0.0%	0.5%	0.0%	0.0%
125. Tsuen Wan	2010	0.00	0.00	0.00	0.00	0.00	150.06	600.05	399.00	525.00	524.00	0.0%	0.0%	0.0%	0.0%	0.0%
126. Tsuen Wan	2011	0.00	0.00	128.76	0.00	189.18	150.39	600.09	446.76	570.00	619.18	0.0%	0.0%	40.5%	0.0%	44.0%
127. Tsuen Wan	2012	0.00	0.00	6.07	0.00	83.44	148.37	593.54	337.07	535.00	535.44	0.0%	0.0%	1.8%	0.0%	18.5%
128. Tsuen Wan	2013	0.00	0.00	45.58	0.00	0.00	149.48	598.00	375.58	542.00	522.00	0.0%	0.0%	13.8%	0.0%	0.0%

129. Tsuen Wan	2014	0.00	0.00	110.21	0.00	138.29	149.33	596.96	391.21	512.00	546.29	0.0%	0.0%	39.2%	0.0%	33.9%
130. Tsuen Wan	2015	0.00	0.00	235.31	0.00	223.33	151.60	603.01	499.31	551.00	625.33	0.0%	0.0%	89.1%	0.0%	55.6%
131. Tsuen Wan	2016	0.00	0.00	69.03	0.00	90.52	145.50	585.09	289.03	458.00	466.52	0.0%	0.0%	31.4%	0.0%	24.1%
132. Tsuen Wan	2017	0.00	0.00	123.17	0.00	113.47	149.58	596.27	333.17	400.00	442.47	0.0%	0.0%	58.7%	0.0%	34.5%
133. Tuen Mun	2007	0.00	0.00	203.36	0.00	152.67	157.23	622.28	745.36	816.00	827.67	0.0%	0.0%	37.5%	0.0%	22.6%
134. Tuen Mun	2008	0.00	0.00	173.75	0.00	200.95	154.26	615.35	760.75	896.00	888.95	0.0%	0.0%	29.6%	0.0%	29.2%
135. Tuen Mun	2009	0.00	0.00	30.71	0.00	118.29	155.09	616.56	733.71	889.00	872.29	0.0%	0.0%	4.4%	0.0%	15.7%
136. Tuen Mun	2010	0.00	0.00	115.12	0.00	111.47	156.98	620.44	712.12	810.00	811.47	0.0%	0.0%	19.3%	0.0%	15.9%
137. Tuen Mun	2011	0.00	0.00	244.07	0.00	296.08	158.41	623.35	825.07	911.00	940.08	0.0%	0.0%	42.0%	0.0%	46.0%
138. Tuen Mun	2012	0.00	1.11	110.36	0.00	195.27	160.07	626.48	717.36	787.00	829.27	0.0%	-0.2%	18.2%	0.0%	30.8%
139. Tuen Mun	2013	1.82	5.80	207.51	0.00	203.40	160.29	626.51	695.51	710.00	771.40	-1.1%	-0.9%	42.5%	0.0%	35.8%
140. Tuen Mun	2014	5.93	17.15	398.39	0.00	293.41	160.29	626.51	772.39	720.00	812.41	-3.6%	-2.7%	106.5%	0.0%	56.5%

141. Tuen Mun	2015	10.02	28.45	447.34	0.00	338.73	160.29	626.51	780.34	699.00	814.73	-5.9%	-4.3%	134.3 %	0.0%	71.2%
142. Tuen Mun	2016	8.70	24.18	450.98	0.00	390.56	160.29	626.51	803.98	746.00	864.56	-5.1%	-3.7%	127.8 %	0.0%	82.4%
143. Tuen Mun	2017	11.59	32.00	340.26	0.00	353.29	160.29	626.51	720.26	666.00	798.29	-6.7%	-4.9%	89.5%	0.0%	79.4%
144. Wanchai	2007	0.00	0.00	98.56	0.00	0.00	126.18	572.97	524.56	698.00	632.00	0.0%	0.0%	23.1%	0.0%	0.0%
145. Wanchai	2008	0.00	0.00	0.00	0.00	104.69	123.36	565.20	435.00	770.00	635.69	0.0%	0.0%	0.0%	0.0%	19.7%
146. Wanchai	2009	0.00	0.00	37.62	0.00	119.07	121.39	558.93	381.62	824.00	624.07	0.0%	0.0%	10.9%	0.0%	23.6%
147. Wanchai	2010	0.00	0.00	66.47	0.00	58.22	123.98	566.76	397.47	775.00	602.22	0.0%	0.0%	20.1%	0.0%	10.7%
148. Wanchai	2011	0.00	0.00	83.78	0.00	34.50	122.61	562.20	395.78	814.00	621.50	0.0%	0.0%	26.9%	0.0%	5.9%
149. Wanchai	2012	0.00	0.00	0.00	0.00	0.00	121.30	557.98	388.00	845.00	637.00	0.0%	0.0%	0.0%	0.0%	0.0%
150. Wanchai	2013	0.00	0.00	27.57	0.00	22.69	122.59	560.12	381.57	799.00	611.69	0.0%	0.0%	7.8%	0.0%	3.9%
151. Wanchai	2014	0.00	0.00	0.00	0.00	83.54	121.84	557.46	374.00	803.00	623.54	0.0%	0.0%	0.0%	0.0%	15.5%
152. Wanchai	2015	0.00	0.00	89.13	0.00	114.54	119.99	551.87	351.13	770.00	612.54	0.0%	0.0%	34.0%	0.0%	23.0%

153. Wanchai	2016	0.00	0.00	73.74	0.00	32.85	121.42	554.78	344.74	752.00	589.85	0.0%	0.0%	27.2%	0.0%	5.9%
154. Wanchai	2017	0.00	0.00	66.67	0.00	79.73	121.28	553.86	294.67	622.00	508.73	0.0%	0.0%	29.2%	0.0%	18.6%
155. Western	2007	0.00	0.00	0.00	0.00	0.00	94.45	565.64	439.00	489.00	525.00	0.0%	0.0%	0.0%	0.0%	0.0%
156. Western	2008	0.00	57.78	0.00	0.00	37.68	99.44	520.90	395.00	554.00	539.68	0.0%	-10.0%	0.0%	0.0%	7.5%
157. Western	2009	0.00	43.37	0.00	0.00	148.67	97.45	528.93	391.00	493.00	597.67	0.0%	-7.6%	0.0%	0.0%	33.1%
158. Western	2010	0.00	60.24	0.00	0.00	125.48	100.16	520.53	354.00	471.00	529.48	0.0%	-10.4%	0.0%	0.0%	31.1%
159. Western	2011	0.00	38.09	0.00	0.00	118.18	99.75	540.19	325.00	520.00	465.18	0.0%	-6.6%	0.0%	0.0%	34.1%
160. Western	2012	0.00	49.21	0.00	0.00	128.09	97.89	523.46	307.00	452.00	456.09	0.0%	-8.6%	0.0%	0.0%	39.1%
161. Western	2013	0.00	35.74	0.00	0.00	160.98	97.65	535.43	316.00	391.00	500.98	0.0%	-6.3%	0.0%	0.0%	47.3%
162. Western	2014	0.00	51.42	0.00	0.00	185.32	99.17	523.17	302.00	388.00	523.32	0.0%	-8.9%	0.0%	0.0%	54.8%
163. Western	2015	0.00	44.60	0.00	0.00	0.00	98.41	527.23	266.00	396.00	365.00	0.0%	-7.8%	0.0%	0.0%	0.0%
164. Western	2016	0.00	46.99	0.00	0.00	114.37	91.46	503.80	251.00	356.00	409.37	0.0%	-8.5%	0.0%	0.0%	38.8%

165. Western	2017	0.00	48.56	0.00	0.00	119.07	94.77	510.77	274.00	391.00	423.07	0.0%	-8.7%	0.0%	0.0%	39.2%
166. Wong Tai Sin	2007	0.00	10.85	0.00	78.53	46.45	141.20	608.86	586.00	693.53	679.45	0.0%	-1.8%	0.0%	12.8%	7.3%
167. Wong Tai Sin	2008	0.00	0.66	0.00	27.68	0.00	135.23	601.86	593.00	685.68	680.00	0.0%	-0.1%	0.0%	4.2%	0.0%
168. Wong Tai Sin	2009	0.00	0.00	41.01	0.00	78.36	132.59	594.17	466.01	726.00	608.36	0.0%	0.0%	9.6%	0.0%	14.8%
169. Wong Tai Sin	2010	0.00	0.00	0.00	0.00	69.37	133.00	594.65	409.00	574.00	525.37	0.0%	0.0%	0.0%	0.0%	15.2%
170. Wong Tai Sin	2011	0.00	0.00	0.00	0.00	112.41	134.54	598.21	398.00	557.00	529.41	0.0%	0.0%	0.0%	0.0%	27.0%
171. Wong Tai Sin	2012	0.00	11.01	0.00	0.00	0.00	134.55	587.12	495.00	612.00	588.00	0.0%	-1.8%	0.0%	0.0%	0.0%
172. Wong Tai Sin	2013	0.00	20.24	0.00	8.66	0.00	136.45	583.43	466.00	580.66	614.00	0.0%	-3.4%	0.0%	1.5%	0.0%
173. Wong Tai Sin	2014	0.00	19.11	0.00	26.15	0.00	137.37	586.27	427.00	527.15	538.00	0.0%	-3.2%	0.0%	5.2%	0.0%
174. Wong Tai Sin	2015	0.00	11.49	0.00	0.00	0.00	139.20	599.10	396.00	569.00	575.00	0.0%	-1.9%	0.0%	0.0%	0.0%
175. Wong Tai Sin	2016	0.00	18.66	0.00	0.00	0.00	141.70	597.56	353.00	475.00	491.00	0.0%	-3.0%	0.0%	0.0%	0.0%
176. Wong Tai Sin	2017	0.00	28.00	0.00	42.45	0.00	143.23	591.73	332.00	455.45	469.00	0.0%	-4.5%	0.0%	10.3%	0.0%

177. Yau Tsim	2007	3.26	0.00	0.00	70.67	0.00	165.39	596.84	520.00	837.67	1316.00	-1.9%	0.0%	0.0%	9.2%	0.0%
178. Yau Tsim	2008	0.00	0.00	0.00	0.00	0.00	168.57	596.19	565.00	708.00	1290.00	0.0%	0.0%	0.0%	0.0%	0.0%
179. Yau Tsim	2009	7.68	0.00	0.00	0.00	0.00	159.59	592.06	540.00	750.00	1158.00	-4.6%	0.0%	0.0%	0.0%	0.0%
180. Yau Tsim	2010	14.61	0.00	0.00	0.00	0.00	155.02	598.62	508.00	670.00	847.00	-8.6%	0.0%	0.0%	0.0%	0.0%
181. Yau Tsim	2011	9.75	0.00	0.00	0.00	0.00	161.56	602.92	545.00	866.00	917.00	-5.7%	0.0%	0.0%	0.0%	0.0%
182. Yau Tsim	2012	18.60	0.00	0.00	0.00	0.00	149.69	594.03	572.00	865.00	1020.00	-11.0%	0.0%	0.0%	0.0%	0.0%
183. Yau Tsim	2013	13.13	0.00	0.00	0.00	0.00	155.40	593.60	461.00	813.00	952.00	-7.8%	0.0%	0.0%	0.0%	0.0%
184. Yau Tsim	2014	7.42	0.00	0.00	0.00	0.00	160.97	592.67	417.00	777.00	806.00	-4.4%	0.0%	0.0%	0.0%	0.0%
185. Yau Tsim	2015	8.93	0.00	34.45	0.00	0.00	159.33	591.77	416.45	861.00	867.00	-5.3%	0.0%	9.0%	0.0%	0.0%
186. Yau Tsim	2016	0.00	0.00	0.00	0.00	0.00	158.71	564.40	364.00	952.00	878.00	0.0%	0.0%	0.0%	0.0%	0.0%
187. Yau Tsim	2017	4.57	0.00	0.00	0.00	0.00	155.21	566.95	394.00	937.00	919.00	-2.9%	0.0%	0.0%	0.0%	0.0%
188. Yuen Long	2007	0.00	0.00	0.00	0.00	0.00	160.29	626.51	989.00	1137.00	1113.00	0.0%	0.0%	0.0%	0.0%	0.0%

189. Yuen Long	2008	1.23	0.00	0.00	0.00	0.00	156.19	618.48	897.00	1042.00	1041.00	-0.8%	0.0%	0.0%	0.0%	0.0%
190. Yuen Long	2009	2.05	0.00	0.00	0.00	28.70	154.32	615.70	847.00	983.00	965.70	-1.3%	0.0%	0.0%	0.0%	3.1%
191. Yuen Long	2010	0.00	0.00	22.30	0.00	188.92	158.55	621.21	831.30	996.00	988.92	0.0%	0.0%	2.8%	0.0%	23.6%
192. Yuen Long	2011	4.40	12.57	94.09	0.00	104.50	160.29	626.51	874.09	991.00	983.50	-2.7%	-2.0%	12.1%	0.0%	11.9%
193. Yuen Long	2012	0.00	0.00	75.45	0.00	0.00	158.47	622.15	871.45	1013.00	1005.00	0.0%	0.0%	9.5%	0.0%	0.0%
194. Yuen Long	2013	0.00	8.66	0.00	0.00	0.00	158.90	615.74	725.00	966.00	1040.00	0.0%	-1.4%	0.0%	0.0%	0.0%
195. Yuen Long	2014	1.59	6.27	220.85	0.00	93.10	160.29	626.51	876.85	971.00	964.10	-1.0%	-1.0%	33.7%	0.0%	10.7%
196. Yuen Long	2015	5.31	17.12	236.45	0.00	35.60	160.29	626.51	880.45	973.00	957.60	-3.2%	-2.7%	36.7%	0.0%	3.9%
197. Yuen Long	2016	15.57	51.90	204.92	0.00	0.00	161.20	624.60	771.92	888.00	905.00	-8.8%	-7.7%	36.1%	0.0%	0.0%
198. Yuen Long	2017	19.71	59.75	307.36	0.00	63.46	160.29	626.51	798.36	819.00	819.46	-11.0%	-8.7%	62.6%	0.0%	8.4%

Notes: i_d = detective-crime ratio; i_u = uniformed police-crime ratio; o_vc = violent crime rate; o_pc = property crime rate; and o_oc = other crime rate.
